# Uncertainty in COVID-19 transmission could undermine our ability to predict long COVID

**DOI:** 10.1098/rsif.2024.0438

**Published:** 2024-12-11

**Authors:** Alexander B. Beams, David J. D. Earn, Caroline Colijn

**Affiliations:** ^1^ Department of Mathematics, Simon Fraser University, 8888 University Dr W, Burnaby, BC V5A 1S6, Canada; ^2^ Department of Mathematics & Statistics, McMaster University, 1280 Main Street West, Hamilton, ON L8S 4K1, Canada

**Keywords:** long COVID, post-acute sequelae of COVID-19, vaccine efficacy, selection, integral equation, ordinary differential equation

## Abstract

As SARS-CoV-2 has transitioned from a novel pandemic-causing pathogen into an established seasonal respiratory virus, focus has shifted to post-acute sequelae of COVID-19 (PASC, colloquially ‘long COVID’). We use compartmental mathematical models simulating emergence of new variants to help identify key sources of uncertainty in PASC trajectories. Some parameters (such as the duration and equilibrium prevalence of infection, as well as the fraction of infections that develop PASC) matter more than others (such as the duration of immunity and secondary vaccine efficacy against PASC). Even if newer variants carry the same risk of PASC as older types, the dynamics of selection can give rise to greater PASC prevalence. However, identifying plausible PASC prevalence trajectories requires accurate knowledge of the transmission potential of COVID-19 variants in the endemic phase. Precise estimates for secondary vaccine efficacy and duration of immunity will not greatly improve forecasts for PASC prevalence. Researchers involved with Living Evidence Synthesis, or other similar initiatives focused on PASC, are well advised to ascertain primary efficacy against infection, duration of infection and prevalence of active infection in order to facilitate predictions.

## 1. Introduction

Post-acute sequelae of COVID-19 (PASC), colloquially long COVID, comprise a wide array of syndromes characterized by systemic inflammation [1]. There are many different hypotheses for the underlying cause of PASC (including the existence of a persistent viral reservoir), but most are consistent with some amount of autoimmunity [2–4]. Some aspects of the condition are becoming more clear: women appear to be at greater risk of PASC than men [5], and immune imprinting from the seasonal coronavirus OC43 may contribute to a dysfunctional immune response against SARS-CoV-2 with autoimmunity the end result [6]. Chronic inflammation in the gut appears to interfere with absorption of tryptophan (a precursor of serotonin), resulting in fatigue and neurologic disorders [7]. Ongoing clinical trials aim to assess the potential for immunologic treatments [2]. In the meantime, PASC may be responsible for contributing nearly 80 disability-adjusted life years per 1000 unhospitalized individuals with COVID-19 infection (greater than 600 disability-adjusted life years (DALYs) for hospitalized cases) [8]. Its future impact on human populations is unclear [9].

Compounding the uncertainty surrounding PASC aetiology is the fact that evolution of new variants will continue [10]. Historically, new scientific findings have translated into practice via the systematic review, which synthesizes and consolidates information from a (sometimes quite large) primary scientific literature into a single document accessible to practitioners in fields such as medicine and public health [11]. However, systematic reviews are time-consuming to produce, and consequently may be too infrequent to be useful [12]. Living Evidence Synthesis (LES) is an alternative process that creates more regular but less comprehensive reports (typically not published in journals, and maintained online) to help ensure that the practice of medicine and public health is in line with current science [12,13]. During the COVID-19 pandemic, LES played a prominent role in developing guidelines and recommendations on a variety of topics, such as mask use and vaccine effectiveness, and was important in the face of variant evolution [13,14]. Despite some duplication of efforts by different groups of researchers, LES were useful for consolidating information from the massive primary literature [14]. Some LES programs continue to assess characteristics of COVID-19, like the likelihood of adverse outcomes of vaccination [15], and vaccine effectiveness against newer variants [16,17]. Without clear treatments useful for PASC, vaccination remains one of the surest ways to control its prevalence. Thus, understanding how vaccines protect against PASC seems essential.

Vaccination protects against PASC by preventing infection in the first place (primary efficacy), and possibly also by reducing the risk of PASC in breakthrough infections (secondary efficacy) [16]. Randomized control trials (RCT) can measure both quantities, but are expensive and time-consuming, so most estimates come from observational data [16]. One method to accomplish this (adapted from studies of influenza vaccine effectiveness) is the test-negative design (TND), a special type of case–control study [18,19]. Although the TND is particularly well suited for estimating influenza vaccine efficacy [19], the TND has also been used for COVID-19 to varying degrees of success [20]. For example, in assessing secondary vaccine effectiveness against PASC, most estimates come from observational studies relying on serological data to indicate prior infection status [16]. However, as we proceed further into the endemic phase of COVID-19, these types of studies may also become increasingly difficult to implement because reduced testing will limit our ability to characterize prior infection status. It should still be possible to estimate a ‘total vaccine efficacy’ confounding the primary and secondary mechanisms, but the utility of such an estimate is questionable.

Predicting future PASC prevalence in the face of continued variant emergence presents several challenges. Under the hypothesis that new variants elicit PASC with the same probability as older ones, selection for more transmissible variants could increase the incidence of infection, and therefore the number of opportunities to develop PASC. However, selection for new variants probably will alter levels of immunity in the population, possibly so that a greater number of exposures are offset by more individuals with protection. Also, uncertainty in the transmission potential of resident types (that is, in the duration of infection, the reproduction number and/or the prevalence of infection by resident types) means that a range of outcomes might be consistent with a new variant’s estimated selection coefficient. Whereas primary efficacy helps characterize the transmission potential of resident types, secondary efficacy against PASC in breakthrough infections informs the baseline probability of PASC. Are these quantities equally important as far as trajectories for PASC are concerned? What other aspects of the disease are important to know?

To address these questions, we use mathematical models to identify the most important sources of uncertainty that affect forecasts of future PASC prevalence. Specifically, we assess the relative importance of primary effectiveness against infection and secondary effectiveness against PASC conditional on infection, and whether an estimate for total vaccine effectiveness against PASC that combines the two mechanisms can be useful for constraining the range of future prevalence. We also assess how uncertainty in characteristics of COVID-19 infection—such as the duration of infection, the duration of immunity, the prevalence of active infection and the baseline probability of developing PASC—affects forecasts. By identifying the most important sources of variability, we identify those aspects of the disease that shape the trajectory of PASC. We do so in the hope of empowering future LES efforts. Faced with a wide array of uncertain characteristics of COVID-19, it can be difficult to identify the most critical aspects of the disease. Our aim here is to identify the ones that are most important to know if predicting PASC prevalence is the goal.

## 2. Methods

We study a simple mathematical model for PASC prevalence incorporating (i) primary efficacy against reinfection, (ii) secondary efficacy against PASC, (iii) PASC duration, (iv) infection duration, (v) underlying prevalence of active infection, and (vi) the emergence and selection of new variants. We will use the model to understand which of these factors affect PASC prevalence the most (and should therefore be measured) as new variants emerge. A central focus is exploring how nonlinear effects from primary efficacy of vaccination interact with other characteristics of the disease to limit PASC prevalence. All simulations of the model are carried out using the deSolve package in R version 4.4.0 [21,22].

### 2.1. Transmission dynamics model

The transmission model incorporates information about primary efficacy against infection, duration of infection, duration of immunity, the transmission rate and the vaccination rate, to calculate the prevalence of active infection. We first describe how transmission dynamics operate without accounting for new variants or PASC, and then modify the model to incorporate PASC.

We ignore vital dynamics because we are interested in studying PASC changes over relatively short spans of time (1−2 years). The population is divided into three parts, consisting of the fraction infected and transmitting to others (I), the fraction with lapsed immunity who are susceptible to infection (S) and the fraction with protective immunity (P). Primary efficacy (η) is the relative reduction in the force of infection experienced by people with protective immunity. Because antibody titres wane over time [23], we assume individuals have temporary immunity (with mean duration $\delta^{-1}$) before lapsing back to susceptible. We assume that all infections transmit equally (at rate β), and last for the same average amount of time ($\gamma^{-1}$), regardless of immunologic status, although there is some evidence to suggest that breakthrough infections in vaccinated people could be less infectious [24]. The equations for the baseline susceptible-infected-protected (SIP) model on which PASC dynamics will be superimposed are


(2.1) $$\begin{array}{rl} \frac{dS}{dt} & =-\beta SI+\delta P-\alpha S, \end{array}\;$$

(2.2) $$\;\begin{array}{rl} \frac{dI}{dt} & =(\beta S+\beta (1-\eta )P)I-\gamma I, \end{array}\;$$

(2.3) $$\;\begin{array}{rl} \frac{dP}{dt} & =\gamma I-\beta (1-\eta )PI-\delta P+\alpha S. \end{array}$$

The basic structure of this model does not change if there is a difference in efficacy (or duration) between immunity from vaccines and infection (appendix A.1), nor if there is a heterogeneous subpopulation at higher risk of infection (appendix A.2). The basic reproduction number is


(2.4) $$\begin{array}{rl} R_{0} & =\frac{\beta }{\gamma }, \end{array}$$

and the real-time reproduction number is


(2.5) $$\begin{array}{rl} R_{t} & =\frac{\beta \left(S+(1-\eta )P\right)}{\gamma }. \end{array}$$

We model vaccination of susceptibles at a constant rate (α), and assume that immunity derived from vaccines is identical to infection-derived immunity because doing otherwise only alters the model slightly. While vaccine-derived immunity is known to differ from infection-derived immunity in terms of antibody specificity and binding affinity for particular proteins, they both confer qualitatively similar types of protection against the same variants, with infection-derived immunity having somewhat higher efficacy [25]. Immunity is ‘leaky’ in the model, meaning that individuals with immunologic protection can experience breakthrough infections, but with a lower probability dictated by the primary efficacy, η. Additionally, breakthrough infections in individuals with immunologic protection may have lower probability of developing PASC compared with infections in susceptible individuals dictated by the secondary efficacy, ϵ (appearing in the next subsection, but not present in equations (2.1)–(2.3)).

### 2.2. Post-acute sequelae of COVID-19 dynamics model

Superimposing PASC dynamics onto the transmission dynamics model requires keeping track of who acquires PASC and how long it persists. We denote the fraction of the population with PASC by the variable u, and the probability susceptible individuals develop PASC symptoms after infection by ϕ. Secondary efficacy (ϵ) is the relative reduction in the rate of PASC acquisition experienced by individuals with protective immunity experiencing a breakthrough infection. We assume that breakthrough infections in vaccinated individuals result in PASC with reduced probability (1−ϵ)ϕ, where ϵ is the secondary efficacy (0<ϵ<1), but that vaccination has no therapeutic effect once PASC manifests [1,2,4].

The total protective efficacy of immunity against PASC accounts for primary efficacy (η) and secondary efficacy (ϵ) according to


(2.6) $$\begin{array}{ccc} & {\text{VE}}_{T}=1-(1-\epsilon )(1-\eta ). & \end{array}$$

Individuals with immunologic protection, P, experience two layers of protection from PASC: by being protected against infection in the first place (primary efficacy, η), and by being less likely to develop PASC if they experience a breakthrough infection (secondary efficacy, ϵ). The average duration of PASC is denoted by $\rho^{-1}$.

While reinfections may increase the risk of PASC [2], it is unclear whether PASC affects susceptibility to reinfection, or viral titres in reinfections. In the absence of this knowledge, we adopt two natural baseline hypotheses: (i) PASC does not affect susceptibility to reinfection, and (ii) PASC does not affect transmission of SARS-CoV-2.

Under these hypotheses, (1−u)S is the probability that an individual is susceptible and does not have PASC, and (1−u)P is the probability that an individual has immunologic protection and does not have PASC. If we assume PASC manifests upon infection or shortly thereafter, and that it resolves at rate ρ, then the rate of change in PASC prevalence in the population is described by the differential equation


(2.7) $$\begin{array}{cccc} & \frac{du}{dt} & =\beta \phi \left((1-u)S+(1-{\text{VE}}_{T})(1-u)P\right)I-\rho u. & \end{array}$$

**Figure 1.**
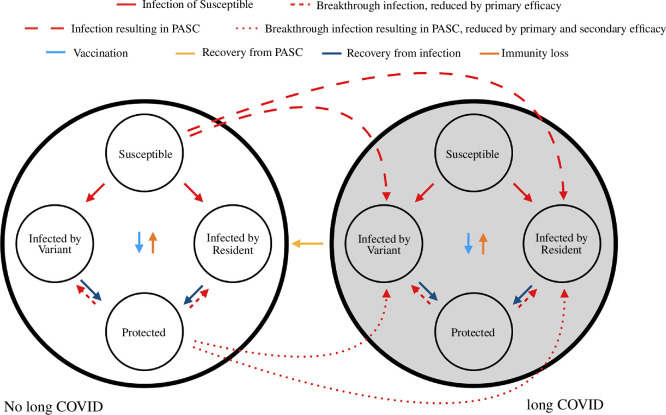
A compartmental model linking PASC to transmission dynamics. A fraction of the population, u, resides in the PASC state represented by the big grey circle. They recover from PASC independent of their infection state. The rest of the population (fraction 1−u) residing in the state without PASC can acquire it with baseline per-infection probability ϕ if they become infected from the susceptible state, or lower probability $(1-{\text{VE}}_{T})\phi$ if they become infected from the protected state. If the transmission dynamics are independent of PASC status, then it is easy to generalize the model to account for delayed onset of PASC some time after acute infection resolves, as well as non-exponential duration of symptoms.

The diagram in figure 1 shows how these assumptions serve to link transmission dynamics with PASC prevalence.

If the dynamics of infection equilibrate, then the steady state PASC prevalence will be


(2.8) $$\begin{array}{l} u^{*}=\frac{\rho^{-1}\beta \phi \left(S^{*}+(1-\epsilon )(1-\eta )P^{*}\right)I^{*}}{\rho^{-1}\beta \phi \left(S^{*}+(1-\epsilon )(1-\eta )P^{*}\right)I^{*}+1}. \end{array}$$

This formula also holds for non-exponetial PASC duration. In appendix A.3, we show that if PASC onset occurs with a distributed delay after infection, and exhibits non-exponential duration, the steady-state PASC prevalence depends on the average duration of PASC (equal to $\rho^{-1}$ under the assumptions we have adopted here), but not on any higher order moments.

### 2.3. Model calibration

In our model, eight parameters control PASC prevalence (table 1). Some of them can be measured or estimated from data, but others cannot. Information that constrains parameter values includes the fraction of cases that develop PASC (F), the prevalence of active infections ($I^{*}$), as well as some epidemiological characteristics such as the duration of infection ($\gamma^{-1}$). There is also information about vaccine efficacy against PASC, but sometimes this information pertains only to secondary efficacy (ϵ), or to total efficacy (${\text{VE}}_{T}$) against PASC, which combines primary and secondary efficacy. We envision that total efficacy will remain relatively easy to estimate in coming years, whereas primary (η) or secondary (ϵ) efficacy may become more difficult to estimate as testing declines. Collectively, these pieces of information place constraints on the model parameters, but do not determine them all.

**Table 1.** Model notation.

	fixed parameters	
parameter	description	baseline value
$\gamma^{-1}$	mean duration of infection	1 week [26–28]
$\delta^{-1}$	mean duration of immunity	1 year [29,30]
α	vaccination rate of susceptibles	1 vaccine per year [31]
$\rho^{-1}$	mean duration of PASC	1 year [2]
η	primary efficacy against reinfection	0−1
ϵ	secondary efficacy against PASC	0−1

	calibration targets	
quantity	description	baseline value
$I^{*}$	prevalence of infection before variant emergence	0.02 [32]
F	fraction of infections developing PASC	0.02 (but may be as high as 0.081−0.110 [8])

	parameters determined by calibration targets and fixed parameters	
parameter	description	baseline value
β	transmission rate	calibrated to $I^{*}$and fixed parameters
ϕ	baseline probability susceptibles develop PASC after infection	calibrated to $I^{*}$, F and fixed parameters

	state variables	
variable	description	possible values
S	proportion susceptible	0−1
I	infection prevalence	0−1
P	proportion protected by immunity	0−1
u	PASC prevalence	0−1

The duration of viral shedding is of the order of 0.5−2 weeks, so in our baseline parametrization we consider the infectious period to be an average of $\gamma^{-1}=1$ week [26–28]. The comprehensive surveillance system in the UK estimates the prevalence of active infection in that country to be 1−4% of the population, with the higher prevalence in winter [32]. A prevalence near 2% and duration of infection of the order of one week means that individuals experience one infection per year, on average. The average duration of immunity probably is not much longer than a year if individuals experience one infection per year (indeed, immune protection appears noticeably diminished by six months [29,30]); we consider a mean duration of immunity of $\delta^{-1}=1$ year in our baseline parametrization (so that roughly 40% of individuals lapse to susceptible after six months). However, the primary efficacy of immunity against reinfection, η, is less well characterized for new variants than it was during the pandemic when vaccines were first introduced, and that in turn makes it difficult to determine the basic reproductive number, $R_{0}$ (equation (2.4)), describing transmission in a completely susceptible population (distinct from the real-time reproductive number describing transmission in the actual population characterized by high levels of pre-existing immunity, equation (2.5)). If $R_{0}$ and η are considered uncertain, then a given prevalence of active infection can be attained through either (i) low primary efficacy and low transmissibility, or (ii) high primary efficacy and high transmissibility. That is, different combinations of the basic reproductive number, $R_{0}$, and the primary efficacy against infection, η, can be consistent with a prevalence of active infection of $I^{*}=0.02$. In appendix A.4, we explain how to obtain $R_{0}$ in terms of η, the other known parameters, and the prevalence of active infection. We consider a scenario where susceptible individuals in the population receive a booster vaccine at a constant rate of α=1 vaccine per year, on average, in line with vaccination trends seen in paediatric settings [31]. In doing so, we are assuming that infection prevalence would be higher in the absence of vaccination.

In considering the value of information pertaining to vaccine efficacy, we examine model scenarios where only primary efficacy is known, only secondary efficacy is known, or the combined total vaccine efficacy against PASC is known (but primary and secondary efficacy are not known separately). We examine the range of PASC prevalences produced under these constraints.

PASC prevalence is difficult to observe directly, but information is available about the fraction of infections that develop PASC: the incidence of PASC as estimated from the US Department of Veterans Affairs (VA) national healthcare database is 81−110 cases per 1000 individuals two years after confirmed infection [8]. In our model, the ratio of PASC incidence and infection incidence is given by


(2.9) $$\begin{array}{cccc} & F & =\frac{\phi \beta SI+\phi (1-\epsilon )\beta (1-\eta )PI}{\beta (S+(1-\eta )P)I}, & \end{array}$$

(2.10) $$\begin{array}{cccc} & & =\frac{\phi (S+(1-\epsilon )(1-\eta )P)}{S+(1-\eta )P}. & \end{array}$$

This ratio is a time-dependent quantity in general, but we use equilibrium values to equate it to values similar to the VA data. If ϵ=0, meaning there is no secondary efficacy against PASC, then F=ϕ, the baseline probability of developing PASC without protective immunity. In simulating PASC prevalence based on particular values of all of the parameters described up to now (in particular, for given values of η and ϵ consistent with a given ${\text{VE}}_{T}$, as well as the equilibrium infection prevalence, $I^{*}$), we choose a value of ϕ consistent with a given fraction of infections developing PASC, F (see appendix A.5).

### 2.4. Emergence of variants

To consider the effect of new variants on PASC prevalence, we augment the basic transmission model to include a new variant emerging to compete with the current one (see appendices A.6 and A.7). We consider variants that compete with the resident for hosts through enhanced transmission (appendix A.6) as well as immune escape (appendix A.7). In either case, we adopt a hypothesis that the new variant has equal propensity to cause PASC as the resident, so that differences in PASC prevalence stem from changes to immunity and incidence of infection occurring due to selection of newer variants in nonlinear fashion. By contrast, variant-specific changes in PASC risk will change prevalence linearly.

In the case of a variant with enhanced transmission, the transmission rate increases over time according to $\beta (t)=\beta (1+s_{0}p(t))$, where β is the transmission rate before the variant appears, $s_{0}$ is the selection coefficient of the variant measured when it is rare, and p(t), the solution to the logistic differential equation, equation A 41, is the frequency of the variant at time t (see appendix A.6 [33]). We treat the case of immune escape similarly, but alter the transmission model to describe an all-or-nothing immunologic mechanism to study temporary disruptions to PASC prevalence in situations when the equilibrium prevalence of active infection remains unchanged (appendix A.7).

## 3. Results

### 3.1. Steady state

We set the average duration of infection in our model to $\gamma^{-1}=1$ week, the equilibrium prevalence of infection to $I^{*}=0.02$, and the fraction of infections developing PASC to F=0.1. If PASC symptoms last for $\rho^{-1}=$ 1 year, then the prevalence of PASC is $u^{*}\approx 0.094$; a duration of $\rho^{-1}=$ 2 years on average nearly doubles the prevalence to $u^{*}\approx 0.172$. In generating trajectories for PASC prevalence in the subsection that follows, we show that pronounced variability in outcomes for PASC prevalence is possible even under optimistic scenarios (where the fraction of infections that develop PASC is F=0.05 or =0.02).

**Figure 2.**
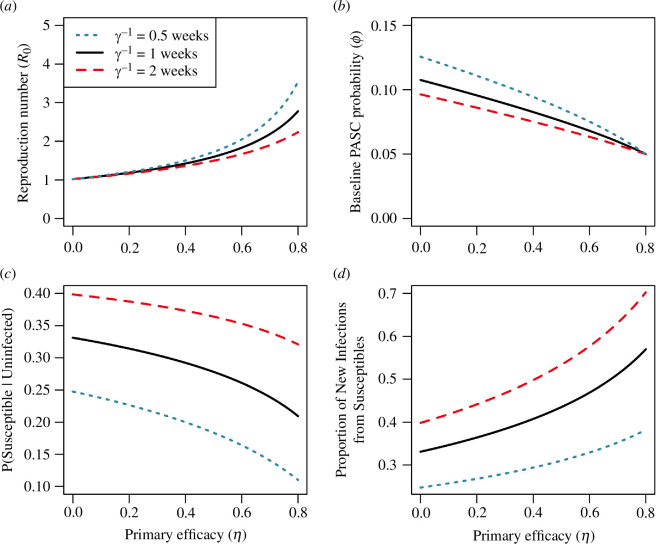
Different combinations of the reproduction number ($R_{0}$), primary efficacy (η), baseline PASC probability (ϕ) and levels of immunity in the population are consistent with calibration targets when prevalence and total vaccine efficacy are known ($I^{*}=0.02$ and ${\text{VE}}_{T}=0.8$). Calibration targets enforce constraints between the reproduction number ($R_{0}$) and primary efficacy (η) (*a*), and between the primary efficacy (η) and the baseline probability of developing PASC (ϕ) (*b*). Because of its role in controlling pathogen transmission, the primary efficacy (η) dictates the fraction of the population that is susceptible (S/(S+P) versus η (*c*)), with stronger immunity concentrating infections within susceptible individuals (S/(S+(1−η)P) versus η (*d*)). Other parameters: vaccination rate of susceptibles, α=1 vaccine per year; mean duration of infection, $\gamma^{-1}=1$ week; mean duration of immunity, $\delta^{-1}=1$ year; fraction of infections developing PASC, F=0.05.

In our model, knowledge of total efficacy against PASC (${\text{VE}}_{T}$) without certainty in primary (η) or secondary efficacy (ϵ) translates into uncertainty in the transmission potential of the virus (figure 2). We assume the prevalence of infection before variant emergence is 2%, and find values of the reproduction number $R_{0}$ that accommodate this calibration target for various values of primary efficacy, η (see table 1). This prevalence of active infection can be attained through a high primary efficacy (η) that limits the transmission of a highly transmissible virus ($R_{0}$), or a virus with low transmission potential and low primary efficacy (figure 2*a*). Likewise, knowledge of ${\text{VE}}_{T}$ introduces a trade-off between primary efficacy (η) and the baseline probability of developing PASC (ϕ) (figure 2*b*). As primary efficacy (η) increases, the proportion of the population susceptible to infection decreases (figure 2*c*), but new infections become increasingly concentrated in susceptible individuals (S/(S+(1−η)P)) (figure 2*c*,*d*). A longer duration of infection ($\gamma^{-1}$) means that the prevalence of infection must be explained by a correspondingly lower reproduction number ($R_{0}$), translating to higher levels of susceptibility in the population (compare the red and blue curves in figure 2).

### 3.2. Primary versus secondary vaccine efficacy

**Figure 3.**
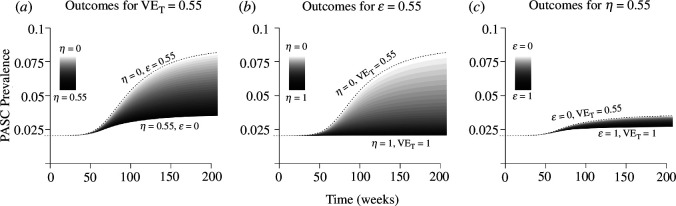
Uncertainty in projected PASC prevalence after variant emergence depends on how estimated total vaccine efficacy (${\text{VE}}_{T}$) is partitioned between primary (η) and secondary (ϵ) efficacy. An estimate of total vaccine efficacy ${\text{VE}}_{T}$ (equation (2.6)) against PASC (*a*) or secondary efficacy ϵ against PASC conditional on breakthrough infection (*b*) gives rise to a wide range of possible changes in PASC prevalence, compared with an estimate for primary efficacy η against infection (*c*). Darker shading corresponds to higher primary efficacy (η). The variant has selection coefficient s=0.1, attained through an increase in the transmission rate, β. The proportion of the population with active infections initially is $I^{*}=0.02$, and the transmission rate β is initially chosen to accommodate $I^{*}$, η and the other epidemiological parameters ($\gamma^{-1}=1$ week, $\delta^{-1}=1$ year, α=1 vaccine per year). The baseline probability of developing PASC, ϕ, is chosen to accommodate F=0.02. PASC duration is modelled by an exponential distribution with mean $\rho^{-1}=1$ year.

We consider an optimistic scenario, where a variant with a higher transmission rate (Δβ>0) has a relatively small selective advantage (s=0.1), and only a small fraction of infections (F=0.02) develop PASC (figure 3). If the total efficacy against PASC is known to be ${\text{VE}}_{T}=0.55$ but primary (η) and secondary (ϵ) efficacy are not known with certainty (equation (2.6)), a wide range of outcomes is attributed to the different combinations of primary and secondary efficacy consistent with the value of ${\text{VE}}_{T}$ (figure 3*a*). At one extreme, if all of the total efficacy against PASC stems from primary efficacy against infection (ϵ=0,η=0.55), PASC prevalence rises to approximately 4% once the variant has replaced the older type, but alternatively if primary efficacy is 0% and all of the protective benefit stems from secondary efficacy against PASC, then the prevalence is expected to rise to just over 8% once the variant has fixed in the population. The per cent change in PASC prevalence responds linearly to $\eta /{\text{VE}}_{T}$.

If secondary efficacy against PASC is known but primary efficacy is not (ϵ=0.55, figure 3*b*), then the range of possible PASC prevalences is even larger, and without ruling out high primary efficacy (η), PASC prevalence could plausibly remain similar to pre-variant levels because immunity might limit transmission of the new variant. Alternatively, if primary efficacy against infection is known (η=0.55), but without any information placing constraints on total efficacy (${\text{VE}}_{T}$) or secondary efficacy (ϵ), PASC prevalence is always limited to a relatively narrow range of 50−100% above the pre-variant baseline (figure 3*c*). Increases in primary efficacy (η) limit transmission to a greater degree, bringing PASC projections associated with low or high secondary efficacy (ϵ=0 or ϵ=1) even closer together (not shown). If vaccine-derived immunity has lower efficacy and shorter duration than infection-derived immunity, the results do not substantially change (appendix A.8, figure 9). Likewise, the presence of subpopulations with elevated risk of infection does not greatly alter the prevalence trajectories for the overall population (appendix A.9, figure 10).

### 3.3. Duration of infection

**Figure 4.**
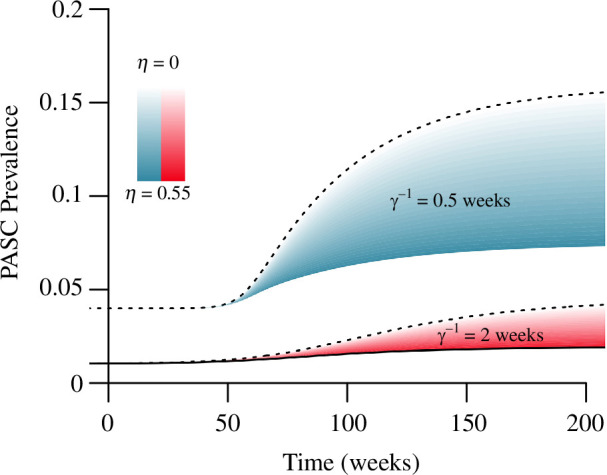
A larger mean duration of infection ($\gamma^{-1}$) restricts PASC increases, and decreases the range of possible outcomes following a novel variant emergence. At longer infection duration, the observed infection prevalence is maintained by a lower incidence, so fewer individuals develop PASC each week (assuming the same fraction of infections (F=0.02) develop PASC). Darker shading corresponds to higher primary efficacy (η). Scenarios assume total vaccine efficacy VE=0.55 as in figure 3*a*, for infection lasting, on average, either $\gamma^{-1}=2$ weeks, or for $\gamma^{-1}=0.5$ weeks. PASC duration is modelled by an exponential distribution with mean $\rho^{-1}=1$ year. All other parameters as in figure 3.

The mean duration of infection is also important for dictating PASC prevalence (figure 4). Our calibration targets impose a fixed prevalence of active infections (2% of the population), as well as the proportion of new infections that develop PASC, but not the prevalence of PASC (table 1). PASC prevalence rises with the number of infections people experience each year (equation (A 30)). Halving the duration of infection from 1 week while holding infection prevalence fixed at $I^{*}=0.02$ means people experience twice as many infections per year, and therefore have twice the probability of developing PASC each year, so the PASC prevalence is higher before and after variant emergence (figure 4). Alternatively, a longer duration of infection of 2 weeks implies people experience one infection every 2 years, so the PASC prevalence is lower. While uncertainty in the duration of infection greatly affects uncertainty in projected PASC prevalence, uncertainty in the duration of immunity (assumed to be $\delta^{-1}$ = 1 year in the baseline parametrization) does not because it has less impact on the number of infections people experience each year (at least for the moderate values of primary efficacy (0<η<0.55) we consider, not shown).

### 3.4. Prevalence of infection

**Figure 5.**
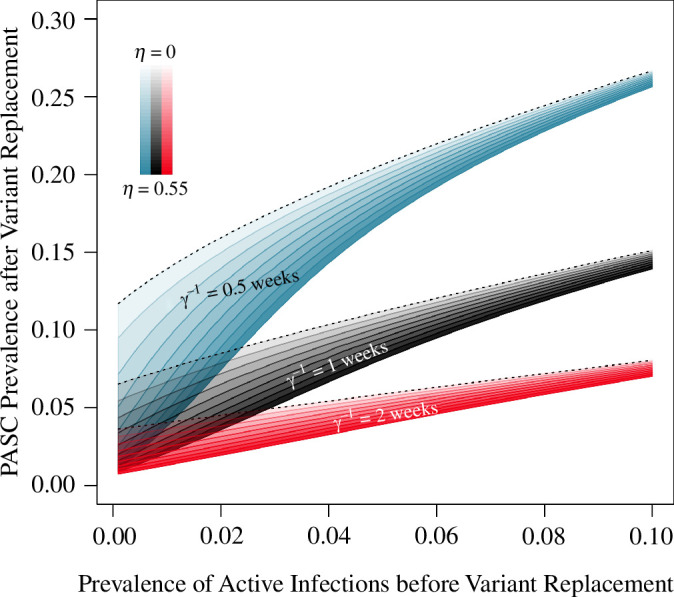
At high infection prevalence, uncertainty in the duration of infection ($\gamma^{-1}$) is more responsible for uncertainty in projected PASC prevalence than uncertainty in total efficacy against PASC (${\text{VE}}_{T}$). At a higher infection prevalence, new variants with a selective advantage will not greatly alter infection incidence, so projections for PASC prevalence are less sensitive to uncertainty in the transmissibility of new variants. At a lower infection prevalence of $I^{*}=0.02$ (consistent with current SARS-CoV-2 estimates; see table 1) uncertainty in the duration of infection and the transmission potential of new variants gives rise to a wide range of PASC prevalence. Total efficacy against PASC is set to 1−(1−ϵ)(1−η)=0.55. The curves represent outcomes at the new steady-state post-variant emergence, with darker shading corresponding to higher primary efficacy (η). All other parameters as in figure 3.

The range of PASC prevalence consistent with ${\text{VE}}_{T}=0.55$ after variant fixation (not long after week 200 in figure 3*a*) depends on the underlying prevalence of active infection before the variant emergence (figure 5). At lower prevalence of active infection, the range of outcomes based on different combinations of primary (η) and secondary (ϵ) efficacy consistent with ${\text{VE}}_{T}=0.55$ is wide, but narrows as infections (and PASC) become more common (figure 5). The calibration targets impose this relationship: because we select the reproduction number ($R_{0}$) to accommodate primary efficacy (η) in a particular scenario (along with the other parameters of the disease; see table 1), a larger prevalence ($I^{*}$) before variant emergence must be explained by a larger reproduction number ($R_{0}$). Because the equilibrium fraction susceptible ($S^{*}$) decreases with the reproduction number ($R_{0}$) (panel (*a*) with panel (*c*) in figure 2), a higher prevalence of active infection implies fewer individuals at high risk ($S^{*}$) for PASC when the variant emerges. Alternatively, a smaller infection prevalence ($I^{*}$) must be accomplished through a lower reproduction number ($R_{0}$) before variant emergence, meaning there are more individuals at high risk ($S^{*}$) who have not yet been infected when the variant does appear. Thus, at low prevalence of active infection, PASC prevalence responds dramatically to the transmissibility of a new variant. At higher prevalence of active infection, the duration of infection ($\gamma^{-1}$) dictates PASC prevalence in the post-variant steady state because the number of infections people experience each year becomes the dominant effect (figure 5).

### 3.5. Variants with a larger $R_{0}$ usually increase long-term post-acute sequelae of COVID-19 prevalence

**Figure 6.**
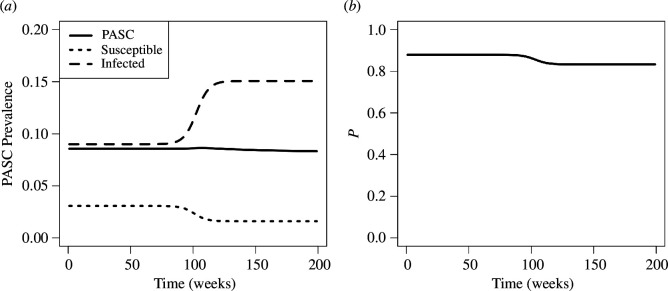
For variant emergence to maintain or slightly reduce PASC prevalence, parameters must take on biologically implausible values. If infections are highly prevalent ($I^{*}=0.09$) while also being very short in duration ($\gamma^{-1}=0.5$ weeks), and if pre-existing immunity is highly efficacious for preventing both reinfection (η=0.65) as well as PASC (ϵ=1), then variants with higher transmission rates can reduce PASC in the long run. PASC duration is modelled by an exponential distribution with mean $\rho^{-1}=1$ year. Other parameters: $\delta^{-1}=52$ weeks, α=1 vaccine per year, F=0.01, s=0.1.

For the range of biologically plausible parameter values we considered, the emergence of a new variant with a higher reproduction number typically increases PASC prevalence. We identified situations where PASC prevalence can decrease, but the parameter values are biologically unrealistic. As an example, if pre-existing immunity has high secondary efficacy against PASC (ϵ≈1) and primary efficacy against reinfection is moderately high, but the virus is so transmissible that the infection prevalence is near 10%, then a variant with an even larger transmission rate (Δβ>0) can decrease PASC in the long run by a small amount (through further depletion of susceptible (S) individuals) (figure 6). Otherwise, variants with a transmission advantage appear likely to increase PASC prevalence because people will experience slightly more infections per year (figures 3–5).

### 3.6. Immune escape variants temporarily increase post-acute sequelae of COVID-19 prevalence

A transmission advantage is not the only way newer variants can replace older ones, however; immune escape is one prominent example [10]. In the idealized situation where a newer variant is identical to the resident in every way, except that it can infect individuals with acquired immunity from the resident virus, while also conferring cross-immunity against the resident, then the newer variant will replace the older one; if it has the same transmission rate, duration of infection, and duration of immunity, it will do so without altering the equilibrium prevalence of infection (see appendix A.7, as well as [10]). However, fluctuations in infections produced by selection can temporarily increase the prevalence of PASC, and it is in these situations that deviation from exponentially distributed PASC duration would be most readily apparent in data.

**Figure 7.**
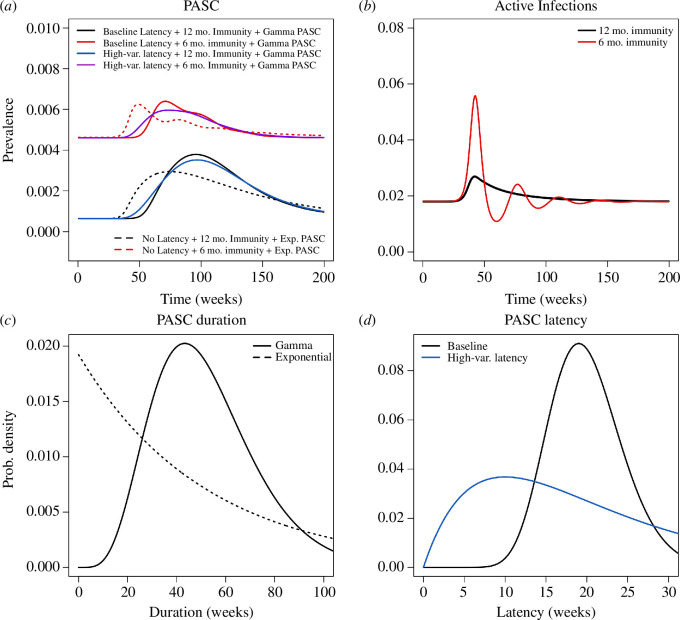
PASC cases can temporarily accrue from an immune escape variant replacing an older type. Model trajectories for PASC prevalence treating the duration of PASC as exponentially distributed with a mean of 1 year (dotted curves in (*a*)) differ from those produced by the integral equation model, which includes Gamma-distributed duration and latency (solid curves in (*a*)). The rise in PASC stems from fluctuations in infection prevalence caused by variant turnover (*b*). In the baseline parametrization, PASC duration is modelled with a Gamma-distribution (shape = 6, scale = 52/6) having the same mean as the exponential model ($\rho^{-1}=1$ year, (*c*)), has a Gamma-distributed latency with a mean of 20 weeks (shape = 20, scale = 1, distribution in (*d*)), and immunity lasts for a mean of $\delta^{-1}=1$ year. Deviations from baseline consider shorter-duration immunity lasting six months (red curves), highly variable latency (Gamma distribution with shape = 2, scale = 10, blue curves), or the combination of both effects (purple curve in (*a*)). Other parameters: initial selection coefficient, $s_{0}=0.4$; $I^{*}=0.018$; α=1 vaccine per year; $\gamma^{-1}=1$ week.

A Gamma-distributed duration of PASC ensures that the rise in PASC prevalence is sustained for a longer amount of time than in the exponential model before returning rapidly to baseline levels (figure 7). Peak PASC prevalence is also higher in the model with Gamma-distributed PASC. Increasing the variability in the latency distribution smoothes PASC prevalence by obscuring fluctuations in incidence, and the relative changes to PASC prevalence become smaller as the average duration of PASC increases (not shown).

### 3.7. Interactions between the duration of infection, the prevalence of infection and the fraction of infections that develop post-acute sequelae of COVID-19

**Figure 8.**
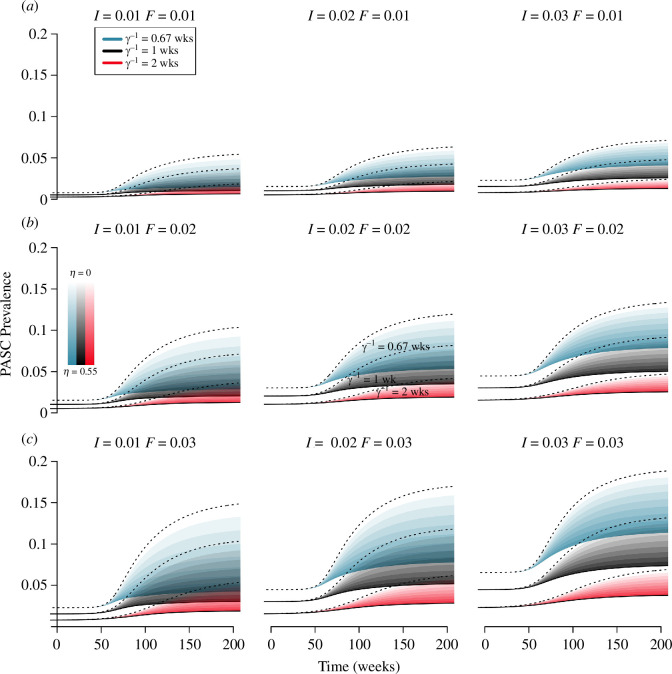
Projections for PASC prevalence are more sensitive to infection duration and the fraction of infections developing PASC than the infection prevalence. All simulations assume a combined efficacy against PASC of ${\text{VE}}_{T}=0.55$, and illustrate the possible outcomes attained through different combinations of primary and secondary efficacy. The prevalence of infection increases across the rows, the fraction of infections developing PASC increases down the columns, and the duration of infection changes within each panel (according to colour). PASC duration is modelled with an exponential distribution with mean $\rho^{-1}=1$ year. Other parameters as in figure 3.

**Figure 9.**
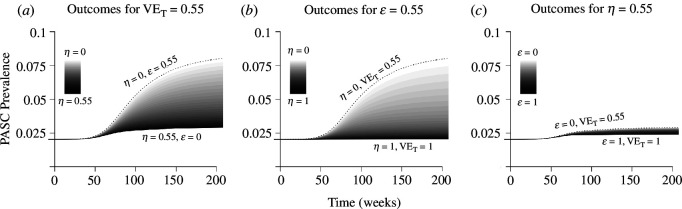
Uncertainty in projected PASC prevalence when efficacy of vaccine-derived immunity is half of the average pre-variant emergence, and the average duration is half that of infection-derived immunity. An estimate of total vaccine efficacy ${\text{VE}}_{T}$ (2.6) against PASC (*a*) or secondary efficacy ϵ against PASC conditional on breakthrough infection (*b*) gives rise to a wide range of possible changes in PASC prevalence, compared with an estimate for primary efficacy η against infection (*c*). Darker shading corresponds to higher primary efficacy (η). Other parameters as in figure 3.

**Figure 10.**
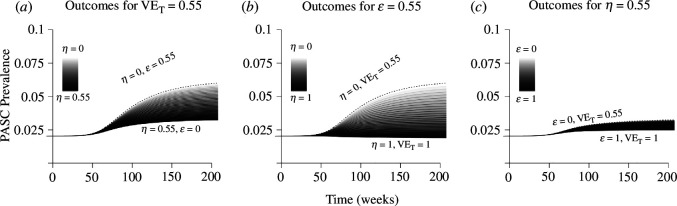
Uncertainty in projected PASC prevalence when 20% of the population is highly susceptible and experiences 80% of new infections. An estimate of total vaccine efficacy ${\text{VE}}_{T}$ (2.6) against PASC (*a*) or secondary efficacy ϵ against PASC conditional on breakthrough infection (*b*) gives rise to a wide range of possible changes in PASC prevalence, compared with an estimate for primary efficacy η against infection (*c*). Darker shading corresponds to higher primary efficacy (η). Other parameters as in figure 3.

We also examine how deviations from a baseline parametrization for duration of infection ($\gamma^{-1}=1$ week), prevalence of infection ($I^{*}=0.02$), and fraction of infections resulting in PASC (F=0.02) interact to affect the prevalence (figure 8; the ‘baseline’ is represented by the black spread of trajectories in the centre panel). For a combined vaccine efficacy against PASC of ${\text{VE}}_{T}=0.55$, the duration of infection, $\gamma^{-1}$, and the fraction of cases developing PASC, F, are more responsible for variation in levels of PASC than the prevalence of infection, $I^{*}$, although the latter always increases the prevalence of PASC.

## 4. Discussion

Uncertainty in primary efficacy of vaccination against reinfection is responsible for producing a wide range of plausible PASC trajectories associated with variant emergence, whereas uncertainty in secondary efficacy against PASC conditional on breakthrough infection is relatively unimportant. Likewise, it is very important to characterize the duration of infection and the fraction of cases that result in PASC, whereas precise estimates for the duration of immunity are less important. LES can help facilitate predictions for PASC prevalence by focusing on primary effectiveness against infection to characterize transmission dynamics in the endemic phase. While consensus on secondary effectiveness is desirable for a number of reasons, it does not greatly facilitate predictions for PASC prevalence.

As we proceed further into the endemic phase and testing becomes less regular, estimating primary efficacy against infection may become more challenging because infection and immunization status will be more obscured than they already are. Studies that measure secondary effectiveness may become rarer for the same reason, but will probably be easier to carry out. On the other hand, studies that measure a combined efficacy against secondary outcomes such as PASC by examining differential vaccine uptake among groups with and without the secondary condition, but without controlling for infections, will be straightforward to carry out. Obviously a measurement of this sort combines different mechanisms of vaccine protection, and seems undesirable for that reason. For developing predictions for PASC, however, our results show that a combined effectiveness estimate may actually be more useful because it places an upper bound on primary effectiveness. Although it is best to know primary effectiveness accurately, given the choice between secondary effectiveness and combined effectiveness, the latter is slightly more useful for predictions and is therefore worth measuring.

Our findings show that strategies for controlling PASC prevalence in the overall population are well aligned with existing strategies for controlling infection. Even though vaccines may confer secondary protection against PASC in breakthrough infections, their optimal allocation is likely to be dictated by their ability to prevent transmission (primary efficacy), which has a nonlinear effect on individual risk. Unless a vaccine is close to 100% effective at preventing PASC, the most effective way to protect people is to reduce their chance of becoming infected in the first place. Future studies intending to identify correlates of PASC risk or duration have the potential to revise forecasts for prevalence. If risk decreases with subsequent reinfections, then PASC prevalence is expected to decrease over time. On the other hand, it is unclear whether PASC alters susceptibility to reinfection, and likewise whether reinfection worsens PASC. If they interact in a positive feedback loop, then cases may become increasingly concentrated within the subset of individuals who experience repeated rounds of reinfection and increasingly morbid symptoms. For future studies aimed to characterize correlates of PASC, it will be important to test the hypothesis that it resolves independent of reinfection. An accurate assessment of the transmission potential of new variants is likely to remain essential for reliable forecasts regardless of the details of interaction, and this will require a reliable understanding of the primary efficacy of vaccination against infection.

Considering a wider array of combined effectiveness measurements for a variety of secondary outcomes besides PASC may help further constrain estimates of primary effectiveness against infection. Any combined effectiveness measurement has the form VE=1−(1−ϵ)(1−η), where ϵ represents secondary effectiveness, and η represents primary effectiveness. Combined vaccine effectiveness estimates for a collection of secondary conditions share the same primary effectiveness against infection, η, but have different secondary effectiveness, ϵ. In the most naive setting, the smallest from a collection of combined effectiveness measurements constitutes the best estimate for η, assuming no other information about primary effectiveness is available. Hence, augmenting LES initiatives studying secondary effectiveness against PASC with complementary ones studying secondary effectiveness against other conditions of interest (unemployment due to complications from COVID-19, death, hospitalization, etc.) may help accomplish the difficult task of constraining estimates for primary effectiveness.

Predicting future PASC prevalence is one goal, but not the only one that benefits from LES. In particular, consensus about secondary vaccine effectiveness against PASC, or other conditions, is important for characterizing vaccine safety, which we have taken as given. We have also considered the probability of PASC to be similar across variants in line with evidence that suggests similar conditions manifest in immunocompromised individuals infected by other kinds of pathogens [2], the assumption being that the probability of PASC onset is greater if immunity has lapsed. Assessing variant-specific secondary effectiveness against PASC is an important test of that assumption.

We developed our model to capture key aspects of COVID-19 as they pertain to PASC. We do not account for coinfection with variants because we do not expect that effect to greatly change the number of infections people experience, and we are not aware of evidence that coinfection plays any particular role in PASC symptoms. We used a leaky model of immunity because it seems consistent with what is currently known about breakthrough infections, although we do not expect results from an all-or-nothing model of immune protection to change our conclusions. We did not explore the consequences of age, sex or other forms of population heterogeneity in detail because the relative importance of transmission over secondary efficacy applies to all demographics. Average prevalence of PASC is useful for understanding the burden on large healthcare systems and economies, but is not a useful descriptor of individuals or in small populations. We did not include asymptomatic infections in our model because it is unclear whether they carry a different risk of PASC to symptomatic infections. We do not account for space in our model because we are not aware of evidence that geography plays a role in PASC, other than its role as proxy for recent transmission.

Simplifying assumptions that facilitated our analysis include (i) that immunity reduces susceptibility to infection, but has no effect on transmission in breakthrough infections; (ii) that PASC occurs after infection with some probability, but does not affect susceptibility to further infection or transmission; (iii) that over time, immunity can lapse, potentially increasing the risk of reinfection and PASC; and (iv) that immunity does not elevate the risk of PASC. The first of these will have only minor impact on our results because our finding that primary efficacy and duration of infection dictate PASC prevalence depends on the average level of transmission in the population. The remaining assumptions could have greater impact on PASC prevalence, but seem to be plausible hypotheses.

In order to investigate plausible scenarios, we reduced a vast parameter space by making simplifying approximations. In the main text, we treat vaccine-derived and infection-derived immunity as identical, but in appendix A.1 and appendix A.8 we show that the effect of a vaccine with lower efficacy and shorter immune duration compared with immunity from natural infection is minor. The primary efficacy (η) becomes a time-dependent quantity, and is reinterpreted as the average primary efficacy of immunity, taken with respect to the relative proportions of people with immunologic protection from vaccines versus people with immunologic protection derived from infection. However, the changes in primary efficacy due to variant emergence are too slight to change our main conclusions. Likewise, the model in the main text considers a homogeneous well-mixed population and is useful for demonstrating the main effects of primary/secondary effectiveness, infection duration, etc. on PASC prevalence in the population as a whole, but is not appropriate for forecasting prevalence experienced by subgroups having higher risk of infection than the general population. We did not include demographics such as sex [5] nor did we include comorbidities which are known correlates of PASC risk [8]. However, we show in appendix A.2 that the effect of a subgroup with higher susceptibility to infection is to introduce time-dependence into the transmission rates for individuals who are susceptible (S) and individuals with immunologic protection (P). Individuals at high risk will experience a larger share of the total PASC burden because they experience more infections per year, but prevalence in the overall population is dictated by the average level of transmission. Even in a situation where PASC cases arise out of a small minority of individuals (appendix A.9), our main conclusions about the relative importance of primary and secondary efficacy hold.

Our study has additional limitations. We treated PASC as a homogeneous secondary condition manifesting temporarily after infection when it instead seems that PASC constitutes a heterogeneous collection of different syndromes, potentially with unique risk factors, and different risks with respect to immune status [2]. The risk of PASC could also increase with the total number of infections a person has experienced in their lifetime [9]. If one wishes to predict PASC prevalence with great accuracy for particular groups of people, then all of these forms of heterogeneity must be accounted for (and measured accurately); our goal, however, has been to highlight the connection between PASC and transmission to support such an endeavour in the future. Although the transmission dynamics of COVID-19 may receive less attention than the more immediate problem of PASC, the former dictate the latter. PASC trajectories produced by the model are more sensitive to the transmission potential of new variants than the ability of vaccines to prevent PASC in breakthrough infections.

Newer variants may carry different risk of PASC, aside from their potential for immune escape or increased transmission potential. In our analysis, we have presented scenarios based on the hypothesis that all variants have equal propensity to elicit PASC, and these show it is difficult to generate reliable predictions unless basic characteristics of infection are known with some precision. Measuring variant-specific PASC risk in real time is likely to be even more difficult for a variety of reasons, not the least of which is the fact that PASC can take months to manifest. As PASC becomes better characterized, it may become clear that the condition depends on the characteristics of individuals’ immune systems to a greater extent than on novel viral characteristics, in which case knowledge of a selection coefficient and the most probable mechanism of variant advantage (e.g. greater transmission potential versus immune escape) will help inform predictions. However, PASC does not manifest without COVID-19 infection in the first place, by definition. Even if infections themselves diminish in stature compared with PASC, their epidemiology retains central importance.

## Data Availability

All codes to run simulations and generate figures are publicly available on Zenodo: [34].

## Appendix A

### A.1. Immunity from vaccines versus infection

The basic form of the model in equations (2.1)–(2.3) is not affected by differences between immune protection derived from infection ($P_{I}$) and protection derived from vaccines ($P_{V}$). If infection trains broader immune recognition than vaccination and can supplement the vaccine, then the interaction between the two forms of immunity may be approximated as dominance of infection-derived immunity over vaccine-derived immunity. In this case, all infections may be treated equal, and recovery from infection always flows into the protection from infection ($P_{I}$) compartment. The model equations are

(A 1)
$$\begin{array}{rl} \frac{dS}{dt} & =-\beta SI+\delta P-\alpha S, \end{array}\;$$

(A 2)
$$\begin{array}{cccc} & \frac{dI}{dt} & =\beta (S+(1-\eta_{P})P_{I}+(1-\eta_{V})P_{V})I-\gamma I, & \end{array}$$

(A 3)
$$\begin{array}{rl} \frac{dP_{I}}{dt} & =\gamma I-\beta (1-\eta_{I})P_{I}I-\delta_{I}P_{I}, \end{array}\;$$

(A 4)
$$\begin{array}{rl} \frac{dP_{V}}{dt} & =\alpha S-\beta (1-\eta_{V})P_{V}I-\delta_{V}P_{V}. \end{array}\;$$

Introducing the variables $P=P_{I}+P_{V}$, $x=P_{V}/P$, along with

(A 5)
$$\begin{array}{cccc} & \eta & =x\eta_{V}+(1-x)\eta_{P}, & \end{array}$$

(A 6)
$$\begin{array}{cccc} & \delta & =x\delta_{V}+(1-x)\delta_{P}, & \end{array}$$

gives rise to equations (2.1)–(2.3), as well as

(A 7)
$$\begin{array}{cccc} & \frac{dx}{dt} & =-\beta ((\eta_{P}-\eta_{V})I+(\delta_{P}-\delta_{V}))x(1-x)+\frac{\alpha S(1-x)}{P}-\frac{x\gamma I}{P}. & \end{array}$$

Thus, differences in vaccine-derived and infection-derived immunity introduce time dependence into the primary efficacy (η) and the duration of immunity (δ), where these quantities now are interpreted as averages taken with respect to the relative proportions of individuals with vaccine-derived and infection-derived immunity.

### A.2. Heterogeneous susceptibility to infection and post-acute sequelae of COVID-19

The basic form of the model in equations (2.1)–(2.3) is also not affected by heterogeneous susceptibility to infection within the population. We assume the fixed population of size N consists of two subpopulations, one with individuals at high risk, $N_{H}$, the other with individuals at low risk, $N_{L}$, and that the proportion of individuals at high-risk, $\sigma =N_{H}/N$, is fixed. If individuals at high risk are more susceptible than the rest of the population by a factor of ζ>1, the model presented in the main text can be modified to

(A 8)
$$\begin{array}{rl} \frac{dS_{L}}{dt} & =-\beta S_{L}(I_{L}+I_{H})+\delta P_{L}-\alpha S_{L}, \end{array}\;$$

(A 9)
$$\begin{array}{rl} \frac{dI_{L}}{dt} & =\beta (S_{L}+(1-\eta )P_{L})(I_{L}+I_{H})-\gamma I_{L}, \end{array}\;$$

(A 10)
$$\begin{array}{rl} \frac{dP_{L}}{dt} & =\gamma I_{L}-\beta (1-\eta )P_{L}(I_{L}+I_{H})-\delta P_{L}, \end{array}\;$$

(A 11)
$$\begin{array}{rl} \frac{dS_{H}}{dt} & =-\zeta \beta S_{H}(I_{L}+I_{H})+\delta P_{H}-\alpha S_{H}, \end{array}\;$$

(A 12)
$$\begin{array}{rl} \frac{dI_{H}}{dt} & =\zeta \beta (S_{H}+(1-\eta )P_{H})(I_{L}+I_{H})-\gamma I_{H}, \end{array}\;$$

(A 13)
$$\begin{array}{rl} \frac{dP_{H}}{dt} & =\gamma I_{H}-\zeta \beta (1-\eta )P_{H}(I_{L}+I_{H})-\delta P_{H}. \end{array}\;$$

where $N_{H}=S_{H}+I_{H}+P_{H}$ and $N_{L}=S_{L}+I_{L}+P_{L}$. Introducing the variables $S=S_{L}+S_{H}$, $I=I_{L}+I_{H}$, $P=P_{L}+P_{H}$, $x=S_{H}/S$, $y=I_{H}/I$ and $z=P_{H}/P$, gives rise to the system

(A 14)
$$\begin{array}{rl} \frac{dS}{dt}= & -\beta (x)SI+\delta P-\alpha S, \end{array}\;$$

(A 15)
$$\begin{array}{rl} \frac{dI}{dt}= & (\beta (x)S+(1-\eta )\beta (z)P)I-\gamma I, \end{array}\;$$

(A 16)
$$\begin{array}{rl} \frac{dP}{dt}= & \gamma I-\beta (z)(1-\eta )PI-\delta P, \end{array}\;$$

(A 17)
$$\begin{array}{rl} \frac{dx}{dt}= & -\beta (1-\zeta )Ix(1-x)-\frac{1-I}{S}\delta x-\delta \frac{yI-\sigma }{S}, \end{array}\;$$

(A 18)
$$\begin{array}{cccc} & \frac{dy}{dt}= & -\beta (1-\eta )(1-\zeta )Iy^{2}-\beta (1-\zeta )Sxy+\eta \zeta \beta Sx+ & \end{array}$$

(A 19)
$$\begin{array}{cccc} & & \beta \left((\eta -1)((I+\sigma )\zeta -I+(1-\sigma ))-\eta S\right)y+\sigma (1-\eta )\zeta \beta & \end{array}$$

with β(x)=xζβ+(1−x)β and β(z)=zζβ+(1−z)β where z=σ−xS−yI. Thus, heterogeneous susceptibility in the population introduces time dependence into the transmission rates, which now differ between individuals who are susceptible (S) and individuals who have immunologic protection (P). The time-dependent transmission rates are now interpreted as averages taken with respect to the relative proportions of high- and low-risk individuals within the S, I and P compartments.

### A.3. Integral equation model for post-acute sequelae of COVID-19

The model in §2.2 treats PASC as manifesting immediately upon infection, so that the prevalence changes according to

(A 20)
$$\begin{array}{cccc} & \frac{du}{dt} & =\beta \phi \left((1-u)S+(1-{\text{VE}}_{T})(1-u)P\right)I-\rho u. & \end{array}$$

This also assumes that PASC duration is exponentially distributed. While these assumptions greatly facilitate our analysis, PASC arises in some individuals long after the acute phase of infection according to a distributed delay. Additionally, once it manifests, the duration of PASC may not be well described by an exponential distribution. Hence, we modify the model for PASC dynamics presented in the main text. We still use the same model for transmission dynamics. In general, the probability that sequelae appear within t weeks of infection could be described by a probability density function (PDF), denoted by h(t), defined for positive durations (t>0). This captures variability in sequelae latency across individuals, but without regard to protective immunity pre-infection. Sequelae duration likewise may not be exponentially distributed. The probability of recovery within t weeks of the first appearance of PASC symptoms could be described by an arbitrary cumulative distribution function (CDF), F(t), defined for positive times t>0. Aside from the restriction that t>0, the distributions F and h might have any form.

At this point, it is convenient to introduce a new variable corresponding to the coefficient of (1−u) appearing in the ODE for u(t) (equation (A 20)),

(A 21)
$$\begin{array}{cccc} & \Sigma & =\beta \phi \left(S+(1-{\text{VE}}_{T})P\right)I. & \end{array}$$

The quantity Σ(1−u) is the incidence of PASC on the assumption that symptoms manifest upon infection. Accounting for a distributed delay between infection and onset of the secondary condition modifies the incidence of PASC at time t through a convolution,

(A 22)
$$\begin{array}{cccc} & \xi (t) & =\int_{0}^{t}\Sigma (\tau )(1-u(\tau ))h(t-\tau )d\tau =\int_{0}^{\infty }\Sigma (t-\tau )(1-u(t-\tau ))h(\tau )d\tau , & \end{array}$$

where h is a PDF capturing describing latency between infection and PASC onset. In the limiting case where the latency is exactly L weeks, h could be a Dirac delta distribution centred at L, h(τ)=δ(τ−L), and then the incidence of PASC at time t, is ξ(t)=Σ(t−L)(1−u(t−L)).

Given the incidence of PASC, ξ, and a CDF for PASC duration, F, the fraction of the population with active secondary conditions at time t becomes

(A 23)
$$\begin{array}{cccc} & u(t) & =\int_{0}^{\infty }\xi (s)(1-F(t-s))ds. & \end{array}$$

Substituting the pieces so far, we obtain

(A 24)
$$\begin{array}{cccc} & u(t) & =\int_{0}^{\infty }\left(\int_{0}^{\infty }\left(1-u(s-\tau )\right)\Sigma (s-\tau )h(\tau )d\tau \right)\left(1-F(t-s)\right)ds. & \end{array}$$

This can be rearranged into a Fredholm integral equation of the second kind for the function u(t),

(A 25)
$$\begin{array}{ccc} & (I+K)u=f, & \end{array}$$

with

(A 26)
$$\begin{array}{rl} Ku & =\int_{0}^{\infty }\int_{0}^{\infty }u(s-\tau )\Sigma (s-\tau )h(\tau )\left[1-F(t-s)\right]d\tau ds, \end{array}$$

(A 27)
$$f=\int_{0}^{\infty }\int_{0}^{\infty }\Sigma (s-\tau )h(\tau )\left[1-F(t-s)\right]d\tau ds,$$

and I the identity. Whenever ‖K‖<1, there is an alternating Neumann series solution

(A 28)
$$\begin{array}{ccc} & u=(I-K+K^{2}-K^{3}+...)f. & \end{array}$$

Equivalently, we can iterate equation (A 24) (a contraction mapping whenever ‖K‖<1,) starting from $u_{0}(t)=0$. The Banach fixed point theorem guarantees iterates will converge to a unique time-dependent solution u(t). This is the method we opt to use in obtaining the trajectories in figure 7. We use the convolve function in R version 4.4.0 [21] to evaluate the integrals.

If the transmission dynamics approach a steady state so that $\lim_{t\to \infty }\;\Sigma (t)=\Sigma^{*}$, the long-term behaviour of u(t), given by $\lim_{t\to \infty }\;u(t)=u^{*}$, satisfies

(A 29)
$$\begin{array}{cccc} & \frac{u^{*}}{1-u^{*}} & =\Sigma^{*}\int_{0}^{\infty }\left[1-F(\tau )\right]d\tau , & \end{array}$$

which clearly does not involve the latency distribution, h.

Because 1−F(t) is a survival probability, the integral in the previous expression is the mean duration of PASC, $\bar{T}$. Thus, the steady-state level of PASC in the population depends only on $\Sigma^{*}$, and the mean duration of PASC, $\bar{T}$, according to

(A 30)
$$\begin{array}{cccc} & u^{*} & =\frac{\Sigma \bar{T}}{1+\Sigma \bar{T}}. & \end{array}$$

Thus at equilibrium, the prevalence of PASC depends on its mean duration, not on any other moments of its distribution. When PASC duration is exponentially distributed, so that $F(t)=1-e^{-\rho t}$, the equilibrium prevalence is the familiar formula $u^{*}=\Sigma^{*}/(\rho +\Sigma^{*})$ (equation (2.8)).

### A.4. Susceptible-infected-protected model steady state

In the SIP model (equations (2.1)–(2.3)), the steady-state number of active infections is a root of the quadratic

(A 31)
$$\begin{array}{ccc} & \left[(1-\eta )\beta^{2}\right](I^{*}{)}^{2}+\left[\beta (\beta (-1+\eta )+(1-\eta )\alpha +\delta +\gamma )\right]I^{*}-\left[(\beta (1-\eta )-\gamma )\alpha -\delta (\beta -\gamma )\right]. & \end{array}$$

Instead of solving for the fixed point in terms of the parameters, we calibrate the model to a known value of $I^{*}$, and assume values for γ, δ and α are known (table 1). This leaves β and η undetermined. Because η is the primary efficacy against infection, it is restricted to values between 0 and 1, but reasonable values for the transmission rate, β, are less obvious *a priori*. However, equation (A 31) is quadratic in β, so we can solve for β in terms of η. Then, we can use equation (2.4) to calculate the values of $R_{0}$ consistent with the assumed values for η, the infection prevalence, and the other parameters.

For fixed values of $I^{*}$, γ,δ and α, but leaving η a free parameter,

(A 32)
$$\begin{array}{cccc} & S^{*}(\eta ) & =(1-I^{*})\frac{\delta }{\beta (\eta )I^{*}+\alpha +\delta }, & \end{array}$$

(A 33)
$$\begin{array}{cccc} & P^{*}(\eta ) & =(1-I^{*})\frac{\beta (\eta )+\alpha }{\beta (\eta )I^{*}+\alpha +\delta }. & \end{array}$$

For fixed duration of infection, of immunity, and a known vaccination rate, a given prevalence of active infection, $I^{*}$ enforces a positive relationship between β and η if they are considered to vary along a single degree of freedom. Thus, a given prevalence of active infection can be explained by a high transmission rate and a high primary efficacy of immunity against reinfection (SIRS dynamics), or by a low transmission rate and little to no primary efficacy against reinfection (SIS dynamics). The bottom-left panel of figure 2 plots the ratio of equation (A 32) relative to the sum of equations (A 32) and (A 33) as a function of η.

The total incidence is β(S+(1−η)P)I. If the dynamics are at steady state (so that vaccination is maintaining rather than disrupting a given level of transmission), then a clinical trial or test-negative study spanning a limited time frame (so that individuals are unlikely to experience more than one infection) measuring vaccine effectiveness against infection obtains a direct estimate of η.

### A.5. Model calibration for ϕ

The primary efficacy against infection, η, controls the relative proportion of $S^{*}$ and $P^{*}$ (equations (A 32) and (A 33), figure 2). Rearranging equation (2.10), we obtain the baseline probability of PASC, ϕ, in terms of η and the fraction of all infections developing PASC, F,

(A 34)
$$\begin{array}{cccc} & \phi (\eta ) & =F\frac{S^{*}(\eta )+(1-\eta )P^{*}(\eta )}{S^{*}(\eta )+(1-{\text{VE}}_{T})P^{*}(\eta )},\;0\leq \eta \leq {\text{VE}}_{T}. & \end{array}$$

If the total vaccine efficacy ${\text{VE}}_{T}$ (equation (2.6)) is assumed known, but the primary and secondary efficacy (η and ϵ) are not, then ϕ could be larger than F, but not smaller. For example, if $\eta ={\text{VE}}_{T}$ and ϵ=0, so that immunity does not confer any additional protection against PASC, then ϕ=F, and all individuals are at equal risk of developing PASC. If, on the other hand, η=0 and $\epsilon ={\text{VE}}_{T}$, individuals in S are at higher risk of PASC than individuals in P, so that ϕ>F.

### A.6. Enhanced transmission

We can augment the basic transmission model from the main text to account for a variant with a different transmission rate. In an idealized situation where the variant ($I_{v}$) and the resident ($I_{r}$) are identical in every way, except that the variant has a slight transmission advantage, the model equations from the main text change to

(A 35)
$$\begin{array}{rl} \frac{dS}{dt} & =-(\beta +\Delta \beta )SI_{v}-\beta SI_{r}+\delta P-\alpha S, \end{array}\;$$

(A 36)
$$\begin{array}{rl} \frac{dI_{v}}{dt} & =(\beta +\Delta \beta )(S+(1-\eta )P)I_{v}-\gamma I_{v}, \end{array}\;$$

(A 37)
$$\begin{array}{rl} \frac{dI_{r}}{dt} & =\beta (S+(1-\eta )P)I_{r}-\gamma I_{r}, \end{array}\;$$

(A 38)
$$\begin{array}{cccc} & \frac{dP}{dt} & =\gamma (I_{v}+I_{r})-(1-\eta )P((\beta +\Delta \beta )I_{v}+\beta I_{r})-\delta P+\alpha S. & \end{array}$$

Setting $dI_{v}/dt$ and $dI_{r}/dt$ simultaneously equal to 0 shows that either $S^{*}+(1-\eta )P^{*}=\gamma /\beta$ and $I_{v}^{*}=0$, or $S^{*}+(1-\eta )P^{*}=\gamma /(\beta +\Delta \beta )$ and $I_{r}^{*}=0$. The first condition cannot be satisfied if $I_{v}(0)>0$ and Δβ>0, so the second condition, where the resident virus is extinct, describes the long-term behaviour of the system whenever the variant is present. Thus, the competitive exclusion principle ensures that whenever there is a steady-state level of infection, the variant drives the resident virus extinct (provided Δβ>0). It is convenient to write $I=I_{v}+I_{r}$ for the total fraction of the population infected, with $p=I_{v}/I$ the probability that an infection is caused by the variant. Then, we see that

(A 39)
$$\begin{array}{cccc} & \frac{dI}{dt} & =\left[(\beta +\Delta \beta )p+\beta (1-p)\right](S+(1-\eta )P)I-\gamma I, & \end{array}$$

(A 40)
$$\begin{array}{rl} & =E[\beta ](S+(1-\eta )P)I-\gamma I, \end{array}\;$$

where 𝔼[β]=[(β+Δβ)p+β(1−p)] is the average transmission rate in the population.

The probability an infection is caused by the variant changes over time according to

(A 41)
$$\begin{array}{cccc} & \frac{dp}{dt} & =\Delta \beta (S+(1-\eta )P)p(1-p). & \end{array}$$

The coefficient of this logistic differential equation is the difference in growth rates between the two variants. As time proceeds, the system approaches $p^{*}=0$ if Δβ<0, $p^{*}=1$ if Δβ>0, or holds steady at $p^{*}=p_{0}$, $0<p_{0}<1$, if Δβ=0.

The selection coefficient is calculated as the ratio of the difference in the growth rates of the two types, divided by the average growth rate,

(A 42)
$$\begin{array}{ccc} & s(t)=\frac{\Delta \beta (S+(1-\eta )P)}{\left(p(\beta +\Delta \beta )+(1-p)\beta )(S+(1-\eta )P\right)}. & \end{array}$$

When the variant is rare (p≪1), the selection coefficient is approximately equal to

(A 43)
$$\begin{array}{cccc} & s_{0} & =\frac{\Delta \beta }{\beta }. & \end{array}$$

If the variant appears when the resident virus is at its equilibrium, then $(S(0)+(1-\eta )P(0))=(S^{*}+(1-\eta )P^{*})=\gamma /\beta$. If we adopt this steady-state approximation to treat the coefficient of the logistic differential equation as a constant, we obtain an approximate solution for the fraction of infections caused by the variant,

(A 44)
$$\begin{array}{cccc} & p(t) & =\frac{p_{0}e^{s_{0}\gamma t}}{1-p_{0}+p_{0}e^{s_{0}\gamma t}}, & \end{array}$$

with $p_{0}\ll 1$ the initial frequency of the variant $I_{v}$ at t=0, and $s_{0}=\Delta \beta /\beta$ the selection coefficient as measured when the variant is rare.

Feedbacks with S and P change the selection coefficient as p changes in the full system, but (A 44) yields a close approximation. Hence, to simulate the emergence and selection of a new variant with a given selection coefficient $s_{0}$, and the impact it has upon PASC prevalence, we simulate the basic transmission model from the main text with time-varying parameters,

(A 45)
$$\begin{array}{cccc} & \beta (t) & =\beta +s_{0}\beta p(t), & \end{array}$$

using (A 44).

### A.7. Immune escape

Whereas the previous model assumes symmetric cross-immunity between variants, we also consider a model that can describe variant replacement solely through immune escape,

(A 46)
$$\begin{array}{cccc} & \frac{dS}{dt} & =-\beta S(I_{r}+I_{v})+\delta (R_{r}+R_{v}), & \end{array}$$

(A 47)
$$\begin{array}{rl} \frac{dI_{r}}{dt} & =\beta SI_{r}-\gamma I_{r}, \end{array}\;$$

(A 48)
$$\begin{array}{cccc} & \frac{dI_{v}}{dt} & =\beta (S+(1-\eta )R_{r})I_{v}-\gamma I_{v}, & \end{array}$$

(A 49)
$$\begin{array}{cccc} & \frac{dR_{r}}{dt} & =\gamma I_{r}-\delta R_{r}-\beta (1-\eta )R_{r}I_{v}, & \end{array}$$

(A 50)
$$\begin{array}{rl} \frac{dR_{v}}{dt} & =\gamma I_{v}-\delta R_{v}, \end{array}\;$$

subject to the initial conditions S(0)=γ/β, $I_{r}(0)=(1-\gamma /\beta )\delta /(\delta +\gamma )-y$, $I_{v}(0)=y$, y≪1, $R_{r}(0)=(1-\gamma /\beta )\delta /(\delta +\gamma )$ and $R_{v}(0)=0$.

This model is the same as that described in [10]. Whenever 0<η<1, the second variant replaces the first, but leaves the equilibrium unchanged. To illustrate the contribution of immune-escape-mediated variant turnover to PASC prevalence, we ignore reinfection in this context, describing immunity through an all-or-nothing mechanism instead. We also assume that the variants carry identical risk of PASC.

Similar to the previous section, we can parametrize η in terms of a selection coefficient. In this case, $p=I_{v}/I$ changes according to

(A 51)
$$\begin{array}{cccc} & \frac{dp}{dt} & =\beta (1-\eta )R_{r}p(1-p). & \end{array}$$

Parametrizing the system in terms of a selection coefficient, $s_{0}$, obtained in the linear regime when p≪1, we have

(A 52)
$$\begin{array}{ccc} & \eta =1-s_{0}\left(\frac{1+\frac{\delta }{\gamma }}{\frac{\beta }{\gamma }-1}\right). & \end{array}$$

Arriving at an approximate solution for the probability an infection is caused by the variant, p(t), is more complicated in this situation, so we simulate the full model to produce the trajectories in figure 7.

In calibrating the reproduction number of this model ($R_{0}=\beta /\gamma$) to an equilibrium value of $I_{r}^{*}$ and a known duration of infection ($\gamma^{-1}$) before variant emergence, it is necessary that

(A 53)
$$\begin{array}{cccc} & \delta & >\frac{I^{*}}{1-I^{*}}\gamma . & \end{array}$$

For $I^{*}=0.02$ and $\gamma^{-1}=1$ week, the average duration of immunity $\delta^{-1}$ cannot be longer than 49 weeks in this model.

### A.8. Results for immunity from vaccines versus infection

Prior to variant emergence, we set the efficacy of vaccine-derived immunity to half the average efficacy, and the average duration of vaccine-derived immunity to half of the average duration. Efficacy of immunity from infection and duration of immunity from infection are chosen so the average efficacy and the average duration match the corresponding averages from the trajectories shown in figure 3. In figure 9, the results are concordant with those shown in figure 3. The time-dependent average primary efficacy (η) and averge rate of immune loss (δ) change over time by a small amount in response to the variant emergence (not shown), so approximating them with constants as in the main text incurs a small amount of error.

### A.9. Results from heterogeneous susceptibility to infection and post-acute sequelae of COVID-19

We examine the impact of population heterogeneity by considering a highly susceptible subpopulation comprising 20% of the total population. We select their susceptibility ζ (see appendix A.2) so that they experience an infection rate four times higher than the rest of the population. The results shown in figure 10 are similar to those shown in figure 3 of the main text. Accounting for population heterogeneity is necessary for developing accurate prevalence trajectories for particular groups, but is less important in generating predictions for the overall population.

## References

[1] Newell KL, Waickman AT. 2022 Inflammation, immunity, and antigen persistence in post-acute sequelae of SARS-CoV-2 infection. Curr. Opin. Immunol. **77**, 102228. (10.1016/j.coi.2022.102228) 35724449 PMC9127180

[2] Altmann DM, Whettlock EM, Liu S, Arachchillage DJ, Boyton RJ. 2023 The immunology of long COVID. Nat. Rev. Immunol. **23**, 618–634. (10.1038/s41577-023-00904-7) 37433988

[3] Merad M, Blish CA, Sallusto F, Iwasaki A. 2022 The immunology and immunopathology of COVID-19. Science **375**, 1122–1127. (10.1126/science.abm8108) 35271343 PMC12828912

[4] Proal AD *et al*. 2023 SARS-CoV-2 reservoir in post-acute sequelae of COVID-19 (PASC). Nat. Immunol. **24**, 1616–1627. (10.1038/s41590-023-01601-2) 37667052

[5] Ortona E, Malorni W. 2022 Long COVID: to investigate immunological mechanisms and sex/gender related aspects as fundamental steps for tailored therapy. Eur. Respir. J. **59**, 2102245. (10.1183/13993003.02245-2021) 34531277 PMC8462012

[6] Herman JD *et al*. 2023 Humoral immunity to an endemic coronavirus is associated with postacute sequelae of COVID-19 in individuals with rheumatic diseases. Sci. Transl. Med. **15**, eadf6598. (10.1126/scitranslmed.adf6598) 37672567 PMC10764151

[7] Wong AC *et al*. 2023 Serotonin reduction in post-acute sequelae of viral infection. Cell **186**, 4851–4867.(10.1016/j.cell.2023.09.013) 37848036 PMC11227373

[8] Bowe B, Xie Y, Al-Aly Z. 2023 Postacute sequelae of COVID-19 at 2 years. Nat. Med. **29**, 2347–2357. (10.1038/s41591-023-02521-2) 37605079 PMC10504070

[9] Saad-Roy CM, Morris SE, Baker RE, Farrar J, Graham AL, Levin SA, Wagner CE, Metcalf CJE, Grenfell BT. 2023 Medium-term scenarios of COVID-19 as a function of immune uncertainties and chronic disease. J. R. Soc. Interface **20**, 20230247. (10.1098/rsif.2023.0247) 37643641 PMC10465195

[10] Otto SP, MacPherson A, Colijn C. 2024 Endemic does not mean constant as SARS-CoV-2 continues to evolve. Evolution. **78**, 1092–1108. (10.1093/evolut/qpae041) 38459852

[11] Grimshaw JM, Eccles MP, Lavis JN, Hill SJ, Squires JE. 2012 Knowledge translation of research findings. Implement. Sci. **7**, 50. (10.1186/1748-5908-7-50) 22651257 PMC3462671

[12] Elliott JH, Turner T, Clavisi O, Thomas J, Higgins JPT, Mavergames C, Gruen RL. 2014 Living systematic reviews: an emerging opportunity to narrow the evidence-practice gap. PLoS Med. **11**, e1001603. (10.1371/journal.pmed.1001603) 24558353 PMC3928029

[13] Elliott J *et al*. 2021 Decision makers need constantly updated evidence synthesis. Nature **600**, 383–385. (10.1038/d41586-021-03690-1) 34912079

[14] Khalil H, Tamara L, Rada G, Akl EA. 2022 Challenges of evidence synthesis during the 2020 COVID pandemic: a scoping review. J. Clin. Epidemiol. **142**, 10–18. (10.1016/j.jclinepi.2021.10.017) 34718121 PMC8550900

[15] Pillay J, Gaudet L, Wingert A, Bialy L, Mackie AS, Paterson DI, Hartling L. 2022 Incidence, risk factors, natural history, and hypothesised mechanisms of myocarditis and pericarditis following COVID-19 vaccination: living evidence syntheses and review. BMJ **378**, e069445. (10.1136/bmj-2021-069445) 35830976 PMC9277081

[16] Shaver N *et al*. 2023 Protocol for a living evidence synthesis on variants of concern and COVID-19 vaccine effectiveness. Vaccine **41**, 6411–6418. (10.1016/j.vaccine.2023.09.012) 37718186

[17] Shaver N. 2023 A living evidence synthesis on variants of concern and covid-19 vaccine effectiveness, mcmaster health forum. Canada: McMaster Health Forum. (doi:20.500.12592/c9qgpj) 10.1016/j.vaccine.2023.09.012 37718186

[18] Sullivan SG, Tchetgen Tchetgen EJ, Cowling BJ. 2016 Theoretical basis of the test-negative study design for assessment of influenza vaccine effectiveness. Am. J. Epidemiol. **184**, 345–353. (10.1093/aje/kww064) 27587721 PMC5013887

[19] Fukushima W, Hirota Y. 2017 Basic principles of test-negative design in evaluating influenza vaccine effectiveness. Vaccine **35**, 4796–4800. (10.1016/j.vaccine.2017.07.003) 28818471

[20] Shi X, Li KQ, Mukherjee B. 2023 Current challenges with the use of test-negative designs for modeling COVID-19 vaccination and outcomes. Am. J. Epidemiol. **192**, 328–333. (10.1093/aje/kwac203) 36446573 PMC10372864

[21] R Core Team: R. 2024 A language and environment for statistical computing. Vienna, Austria: R Foundation for Statistical Computing. See https://www.R-project.org.

[22] Soetaert K, Petzoldt T, Setzer RW. 2010 Solving differential equations in R: package deSolve. J. Stat. Softw. **33**, 1–25. (10.18637/jss.v033.i09)20808728

[23] Chia WN *et al*. 2021 Dynamics of SARS-CoV-2 neutralising antibody responses and duration of immunity: a longitudinal study. Lancet Microbe **2**, e240–e249. (10.1016/S2666-5247(21)00025-2) 33778792 PMC7987301

[24] Abu-Raddad LJ *et al*. 2022 Relative infectiousness of SARS-CoV-2 vaccine breakthrough infections, reinfections, and primary infections. Nat. Commun. **13**, 532. (10.1038/s41467-022-28199-7) 35087035 PMC8795418

[25] Yu Y, Esposito D, Kang Z, Lu J, Remaley AT, De Giorgi V, Chen LN, West K, Cao L. 2022 mRNA vaccine-induced antibodies more effective than natural immunity in neutralizing SARS-CoV-2 and its high affinity variants. Sci. Rep. **12**, 2628. (10.1038/s41598-022-06629-2) 35173254 PMC8850441

[26] Byrne AW *et al*. 2020 Inferred duration of infectious period of SARS-CoV-2: rapid scoping review and analysis of available evidence for asymptomatic and symptomatic COVID-19 cases. BMJ Open **10**, e039856. (10.1136/bmjopen-2020-039856) PMC7409948 32759252

[27] van Kampen JJA *et al*. 2021 Duration and key determinants of infectious virus shedding in hospitalized patients with coronavirus disease-2019 (COVID-19). Nat. Commun. **12**, 267. (10.1038/s41467-020-20568-4) 33431879 PMC7801729

[28] Keske Ş *et al*. 2023 Duration of infectious shedding of SARS-CoV-2 Omicron variant and its relation with symptoms. Clin. Microbiol. Infect. **29**, 221–224. (10.1016/j.cmi.2022.07.009) 35853589 PMC9287585

[29] Feikin DR *et al*. 2022 Duration of effectiveness of vaccines against SARS-CoV-2 infection and COVID-19 disease: results of a systematic review and meta-regression. The Lancet **399**, 924–944. (10.1016/S0140-6736(22)00152-0) PMC8863502 35202601

[30] Walsh KA *et al*. 2024 Duration of protective immunity following COVID‐19 vaccination of individuals with underlying health conditions: a rapid review. Rev. Med. Virol. **34**, e2504. (10.1002/rmv.2504) 41063671

[31] Ryan GW, Goulding M, Beeler AL, Nazarian BL, Pbert L, Rosal MC, Lemon SC. 2023 Trends in COVID-19 vaccine administration across visit types in a safety net pediatric practice during the first year of authorization. Front. Pediatr. **11**. (10.3389/fped.2023.1227115) PMC10657821 38027270

[32] UK Health Security Agency. 2023 Winter Coronavirus (COVID-19) Infection Study: estimates of epidemiological characteristics. See https://www.gov.uk/government/statistics/winter-coronavirus-covid-19-infection-study-estimates-of-epidemiological-characteristics-england-and-scotland-2023-to-2024/winter-coronavirus-covid-19-infection-study-estimates-of-epidemiological-characteristics-21-december-2023#data-sources.

[33] Day T, Gandon S. 2007 Applying population-genetic models in theoretical evolutionary epidemiology. Ecol. Lett. **10**, 876–888. (10.1111/j.1461-0248.2007.01091.x) 17845288

[34] Beams AB. Long COVID model. Zenodo (10.5281/zenodo.13901216)

